# Early Warning for Ovarian Diseases Based on Plasma Non-esterified Fatty Acid and Calcium Concentrations in Dairy Cows

**DOI:** 10.3389/fvets.2021.792498

**Published:** 2021-12-08

**Authors:** Yuxi Song, Jiaxin Cheng, Hang Yu, Zhijie Wang, Yunlong Bai, Cheng Xia, Chuang Xu

**Affiliations:** Heilongjiang Provincial Key Laboratory of Prevention and Control of Bovine Diseases, College of Animal Science and Veterinary Medicine, Heilongjiang Bayi Agricultural University, Daqing, China

**Keywords:** dairy cows, inactive ovaries, follicular cysts, luteal cysts, early warning

## Abstract

Inactive ovaries (IO) and ovarian (follicular or luteal) cysts (FC or LC) are two common ovarian diseases leading to infertility in dairy cattle. Both disorders are associated with altered metabolites and hormones. There are currently no known effective biomarkers that can be used for early diagnosis of ovarian diseases. The purpose of this study was to identify the plasma biomarkers of ovarian diseases in Holstein dairy cows that facilitate an early diagnosis of the diseases and control its progression. The experiment was performed from 3 weeks postpartum and last for 7 weeks. Seventy-six multiparous Holstein cows (mean age, 4.36 years; weight, 635.63 kg) were divided into healthy control group (HC, *n* = 22), FC group (*n* = 18), LC group (*n* = 18) and IO group (*n* = 18) by rectal palpation or ultrasonography during the last 2 weeks before trial end. Blood was collected via tail vein for measurement of plasma energy metabolites, liver function indicators, minerals, and hormones at 3 and 8 weeks postpartum. Data were analyzed by Mann-Whitney U, Kruskal-Wallis, Spearman correlation, binary logistic regression analysis and receiver operating characteristic analysis, where applicable. At 8 weeks postpartum, FC cows had a more severe body condition score loss and these had greater levels of non-esterified fatty acids (NEFA) and estradiol, and lesser levels of alanine aminotransferase (ALT), progesterone and insulin-like growth factor 1 (IGF-1) levels than HC cows (*P* < 0.05). LC cows had a lower milk yield, higher NEFA and progesterone levels, and lower calcium, phosphorus and magnesium levels than HC cows (*P* < 0.05). IO cows had a lower body condition score, higher NEFA levels, and lower ALT, calcium, phosphorus, magnesium, estradiol, progesterone and IGF-1 levels than HC cows (*P* < 0.05). At 3 weeks postpartum, cows with ovarian diseases had greater (*P* < 0.05) concentrations of NEFA, and lesser concentrations of ALT, calcium, phosphorus and IGF-1 than HC cows. Early warning values for ovarian diseases were plasma NEFA concentrations >0.50 mmol/L, or calcium concentrations <2.02 mmol/L. Therefore, plasma NEFA and calcium could be used as early-warning indicators for ovarian diseases in dairy cows.

## Introduction

Selection for milk yield in dairy cows in the last 70 years has resulted in a significant increase in milk yield and concomitant reduction in fertility (1). Inactive ovaries (IO) and ovarian cysts are two major causes of infertility in dairy cows (2, 3). Historically, IO has been defined as growth of follicles only to the stage of follicular wave emergence, that is, up to ~8 mm diameter, after which, growth stops (4), while ovarian cysts (follicular or luteal) are defined as anovulatory ovarian structures with a cavity >20 mm in diameter in the absence of a corpus luteum (5). Notably, the difference between follicular and luteal cysts (LC) is that the wall is <3 mm in follicular cyst (FC) and >3 mm in LC (6).

About 20% of dairy cows in anestrus by the start of breeding programs (7) or by 63 days after calving (8) were involved in the IO. Around the same time, the incidence of ovarian cysts may vary from 2.7 to 15.1% (9, 10) or from 6 to 30% (11, 12). Both ovarian diseases may increase calving to conception interval, calving interval, calving to first service interval and days open. In addition, cows affected may be at greater risk of being culled because of poor reproductive performance (13), thereby causing vast economic losses to the dairy industry (2). Therefore, it is crucial to discover novel biomarkers for early detection of ovarian diseases and monitoring of disease progression.

The transition period is one of the most challenging periods in dairy cows and encompasses the 3 wk prior to and 3 wk after parturition (14, 15). During this period, cows undergo drastic adaptations as regards the metabolism of glucose, fatty acids and minerals (14, 16). Despite this, parturition and the onset of lactation put an enormous physiological stress on the cow's homeostatic processes (17). Further, pregnancy and lactation are recognized as inducing remarkable physiological and metabolic adaptations in dairy cows essential for a good reproductive and productive performance (18–20). Despite the action of homeostatic mechanisms to maintain blood parameters within physiologic levels, changes in metabolites and hormones occur as a result of increased metabolic demands in lactating animals. These changes are not necessarily indicative of diseases but make animals physiologically unstable and more susceptible to a number of metabolic diseases at this stage than during other life periods compromising productivity (21).

High producing dairy cows often undergo a period of negative energy balance (NEB) during the first weeks of lactation (22). The NEB status in early lactation is characterized by alterations in blood metabolite and hormone profile, which is of critical importance to subsequent health and fertility (14, 23). Altered patterns of blood biomarkers have been used as diagnostic indicators of some diseases for a few decades. For example, elevated blood β-hydroxybutyrate prepartum (24–26), serum non-esterified fatty acids (NEFA) prepartum (26–28), serum calcium the week before and soon after calving (26–29) have been used to predict postpartum diseases.

However, it should be noted that there are currently no effective early markers available for early diagnosis of ovarian diseases in dairy cows. Although an reliable diagnosis of ovarian diseases currently employs a combination of trans-rectal palpation, trans-rectal ultrasonography and plasma progesterone assay (30). Unfortunately, once diagnosed, it is too late to intervene effectively. Thus, the objective of this study was to identify the plasma biomarkers of the ovarian diseases in Holstein dairy cows that facilitate an early diagnosis of the diseases and control its progression.

## Materials and Methods

### Ethics

The study protocol was approved by the Ethics Committee (SY201909005) on the Use and Care of Animals of Heilongjiang Bayi Agricultural University (Daqing, China).

### Animals

This prospective observational experiment was conducted on a commercial farm in Heilongjiang Province, China, from September 2019 to January 2020. The experiment was performed from week 3 postpartum and last for 7 weeks. Seventy-six multiparous Holstein cows (parity 2–5) without oestrus synchronization were divided into healthy control group (HC, *n* = 22; mean age, 4.25 ± 0.94 years; mean weight, 634.50 ± 22.90 kg), FC group (*n* = 18; mean age, 4.63 ± 0.19 years; mean weight, 652.28 ± 36.25 kg), LC group (*n* = 18; mean age, 4.03 ± 0.59 years; mean weight, 635.34 ± 12.95 kg) and IO group (*n* = 18; mean age, 4.54 ± 0.87 years; mean weight, 620.64 ± 34.41 kg) by rectal palpation or ultrasonography during the last 2-week period. Inactive ovaries and ovarian cysts were recorded as ovarian disorders. All cows were suffering no other clinical disorders. The subject animals were maintained in free-stall housing with continuous access to fresh water and were milked three times per day. Total mixed rations during early lactation were formulated in accordance with the 2001 US National Research Council standards. The total mixed rations consisted of 8–9 kg of concentrate, 19 kg of silage, 3.5–4.0 kg of hay, and 350 g of fat. Feed analysis showed 55.60% of dry matter, 16% of crude protein, 7.322 MJ·kg^−1^ net lactation production, 5.60% of fat, 39.10% of neutral detergent fiber, 20.30% of acid detergent fiber, 180 g of calcium, and 116 g of phosphorus.

### Ovarian Ultrasonography

Ovarian ultrasonographic examinations were performed in all cows by using a DP-2,200 VET ultrasonograph (Mindray Biomedical Electronics) as described previously. Briefly, Healthy control group were selected from cows with normal corpus luteum and/or large follicles (>10 mm) in two examinations (10 days apart). Inactive ovaries group were selected from cows without ovarian cysts, persisted corpus luteum or follicle, and normal corpus luteum and/or large follicles (>10 mm) (2). Two ultrasonographic examinations of the ovaries, approximately 7 d apart, reveal no substantial changes in the follicular structures (31). Follicular cysts were characterized by a size of 3–5 cm, a thin wall ≤ 0.3 cm, duration of maintenance of at least 10 days, and progesterone concentration < 1 ng/mL, whereas luteal cysts were typified by a diameter of 3–5 cm, a cavity filled with liquid, a thick luteinized wall with a thickness >0.3 cm that persisted for at least 14 days, and progesterone concentration >1 ng/mL (32, 33).

### Data Collection

Age, parity, weight and milk yield were collected from the Afitag pedometer (Afimilk 0418A09QPDX, Kibbutz Afikim, Israel) of the cattle farm. Body condition score (BCS) was weekly done by 2 trained farm veterinarian using the established 5-point method ranged from 1 to 5 with 0.25 unit intervals (34). Body condition score loss was calculated as the difference between the BCS on wk 3 and wk 8.

### Sample Collection

At 3 wk and 8 wk postpartum, before milking and fasting in the morning, 10 mL of blood was collected from the jugular vein into an anticoagulant tube (BD, Franklin Lakes, NJ) containing sodium heparin. Anticoagulated blood was centrifuged at 1,500 × g for 5 min and the supernatant was placed into a 1.5 mL Eppendorf tube. Then, the supernatant was centrifuged at 12,000 × g for 5 min and 500 μL of plasma was transferred into a 1.5 mL Eppendorf tube and stored at −80°C for biochemical analysis, radioimmunoassay and ELISA.

### Plasma Analysis

β-hydroxybutyrate, NEFA, glucose, triglycerides, total cholesterol, urea nitrogen, aspartate aminotransferase, alanine aminotransferase (ALT), γ-glutamyltransferase, total protein, albumin, globulin, total bilirubin, calcium, phosphorus and magnesium in plasma were analyzed using commercial biochemical assay kits (Mindray Biomedical Electronics Co. Ltd, Shenzhen, China). All metabolite concentrations were quantified using a Mindray BS-830S fully automatic biochemistry analyzer (Mindray Biomedical Electronics Co. Ltd, Shenzhen, China). All measurements were executed according to the manufacturer's instructions.

Estradiol, progesterone, insulin and growth hormone concentrations were measured using four commercial kits with the same lot number (Xinfan Biotechnology Co. Ltd, Shanghai, China) and the kit manufacturer's validated radioimmunoassay procedures. Assay sensitivities were 2 pg/ml (estradiol), 0.2 ng/ml (progesterone), 2 μIU/ml (insulin) and 10 ng/ml (growth hormone). Intra-assay coefficients of variation is >10%, inter-assay coefficients of variation is >15%.

The quantities of insulin-like growth factor 1 (IGF-1) were detected using a bovine-specific ELISA kit purchased from Xinfan Biotechnology Co., Ltd. (Shanghai, China), in accordance with the manufacturer's instructions.

### Statistical Analysis

Statistical analyses were performed using SPSS 26.0 (IBM, New York, NY) software. Since the data did not fit a normal distribution, non-parametric tests (Kruskal-Wallis rank test or Mann-Whitney U-test) were used. Group differences were first tested with the Kruskal-Wallis rank test and if significant they were analyzed further with the Mann-Whitney U-test. Data were expressed as mean ± SD. Differences between HC and ovarian diseases groups were compared by the Mann-Whitney U-test. Data were presented as average ± SEM. Correlation between plasma biomarkers and ovarian diseases were explored using Spearman rank correlation coefficients. To predict diseases, binary logistic regression models were created. We finally used the Relative Operating Characteristic (ROC) curve to test the significance of the logistic regression models. Statistical significance is set as *P* < 0.05.

## Results

**Figure 1:**
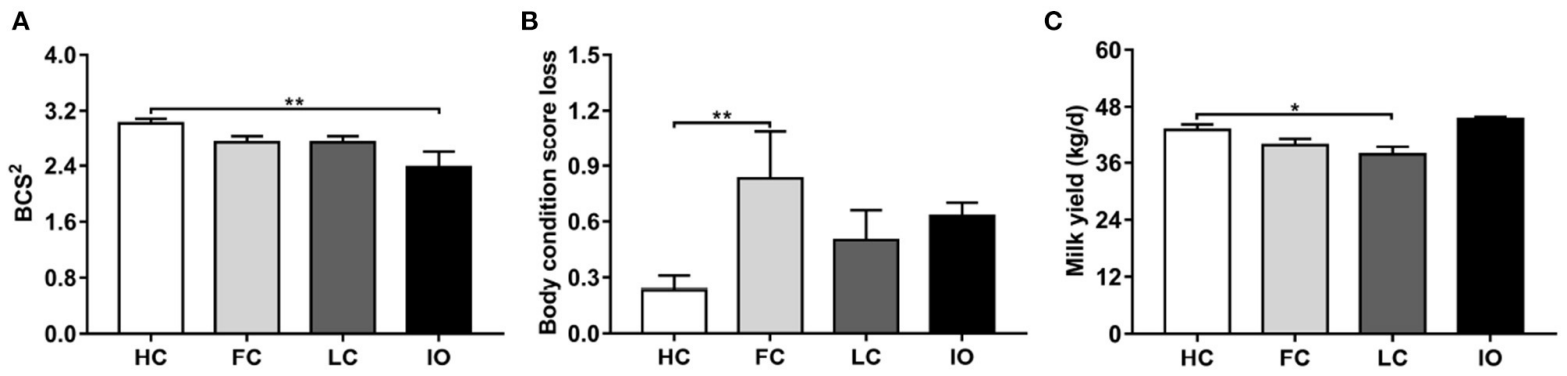
**(A–C)** Comparison of general data between the follicular cysts (FC, *n* = 18) group, luteal cysts (LC, *n* = 18) group, and inactive ovaries (IO, *n* = 18) group and healthy control (HC, *n* = 22) group, respectively. ^*^P < 0.05, ^**^P < 0.01 vs. healthy control group. BCS^2^, body condition score during the 8th week postpartum.

Table 1 shows the distribution of age, parity, BCS, and milk yield for all 76 cows. There are significant differences in the BCS^2^ and milk yield among the four groups (*P* < 0.05). Body condition score loss showed a marginal significant difference (*P* = 0.051). The results were further analyzed by comparing the different groups of diseased and healthy animals (Figure 1). The mean BCS^2^ was significantly lower in the IO group compared to the HC group (2.38 ± 0.22 vs. 3.02 ± 0.07, *P* < 0.01) (Figure 1A). Body condition loss was significantly higher in FC compared with HC groups (0.83 ± 0.26 vs. 0.23 ± 0.08, *P* < 0.01) (Figure 1B). Milk yield was significantly lower in LC than in HC groups (37.77 ± 1.79 kg/d vs. 45.45 ± 0.44 kg/d, *P* < 0.05) (Figure 1C).

**Table 1:** Kruskal-Wallis test of general data among the four groups (*n* = 76).

**Parameters**	**Mean**	**SD**	**0**	**25%**	**50%**	**75%**	**100%**	**χ^2^**	***P*-value**
Age	4.3	0.82	3	4	4.1	4.9	6.4	3.027	0.387
Parity	3.05	0.77	2	3	3	3	5	2.017	0.569
^BCS^1^^	3.28	0.43	2.5	2.75	3.5	3.5	4	3.914	0.271
^BCS^2^^	2.87	0.36	2	2.75	2.75	3.25	3.5	9.686	0.021
BCS loss	0.41	0.45	−0.5	0	0.25	0.75	1.25	7.761	0.051
Milk yield (kg/d)	42.07	5.05	33.86	37.96	43.29	46.21	50.1	9.362	0.025

BCS^1^ *, body condition score during the third week postpartum*;

BCS^2^ *, body condition score during the 8th week postpartum; BCS loss, body condition score loss*.

**Figure 2:**
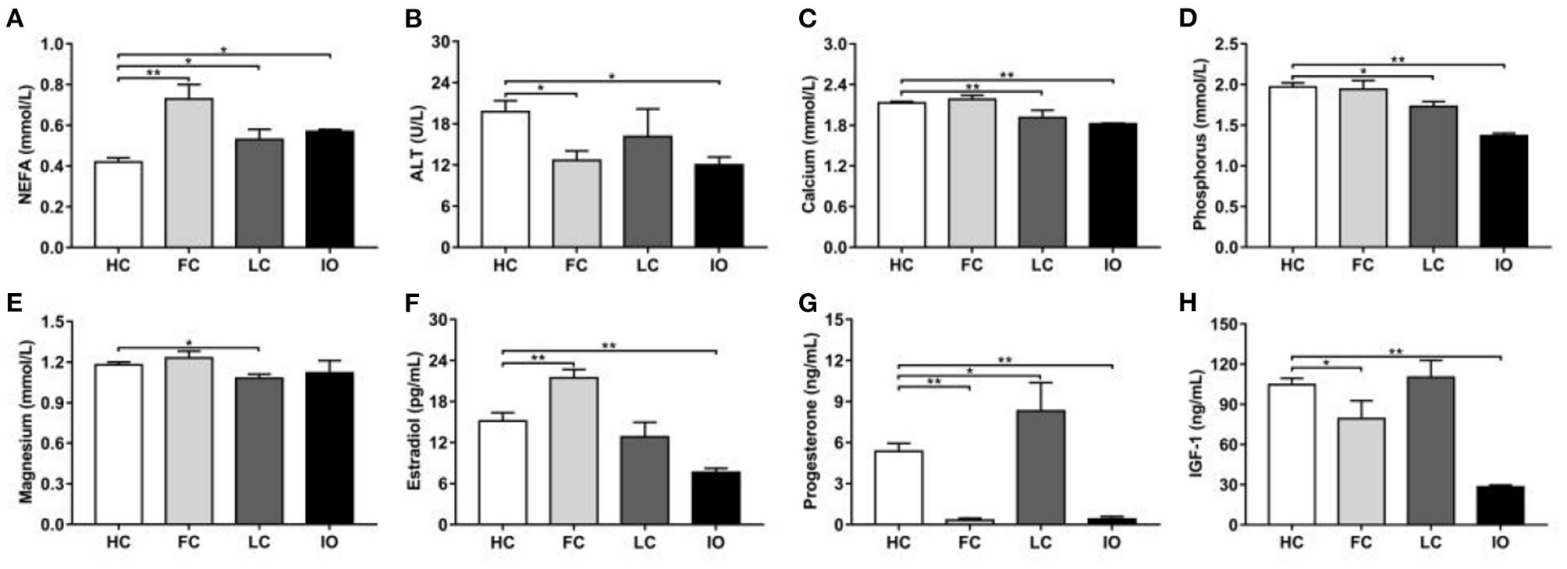
**(A–H)** Comparison of plasma biochemical indicators between the follicular cysts (FC, *n* = 18) group, luteal cysts (LC, *n* = 18) group, and inactive ovaries (IO, *n* = 18) group and healthy control (HC, *n* = 22) group, respectively. Note: ^*^P < 0.05, ^**^P < 0.01 vs. healthy control group. NEFA, non-esterified fatty acids; ALT, alanine aminotransferase; IGF-1, insulin-like growth factor 1.

The same analysis was also performed on plasma biochemical indexes. There were significant differences (*P* < 0.05) in plasma NEFA, ALT, calcium, phosphorus, magnesium, estradiol, progesterone, and IGF-1 levels among groups (Table 2). Figure 2 shows the results of the subgroup analyses. Plasma NEFA levels were significantly higher in FC, LC, and IO than in HC cows (0.73 ± 0.07, 0.53 ± 0.05, and 0.57 ± 0.01 vs. 0.42 ± 0.02 mmol/L, *P* < 0.05) (Figure 2A). Plasma ALT levels were significantly lower in FC and IO than in HC cows (12.67 ± 1.38, and 12.00 ± 1.15 vs. 19.77 ± 1.60 U/L, *P* < 0.05) (Figure 2B). Plasma calcium levels were significantly lower in LC and IO than in HC cows (1.91 ± 0.11, and 1.82 ± 0.01 vs. 2.13 ± 0.02 mmol/L, *P* < 0.01) (Figure 2C). Plasma phosphorus levels were significantly lower in LC and IO than in HC cows (1.73 ± 0.06, and 1.37 ± 0.03 vs. 1.97 ± 0.05 mmol/L, *P* < 0.05) (Figure 2D). Plasma magnesium levels were significantly lower in LC than in HC cows (1.08 ± 0.03 vs. 1.18 ± 0.02 mmol/L, *P* < 0.01) (Figure 2E). Plasma estradiol levels were significantly higher in FC cows and lower in IO cows than in HC cows (21.45 ± 1.25, and 7.63 ± 0.61 vs. 15.14 ± 1.21 pg/mL, *P* < 0.01) (Figure 2F). Plasma progesterone levels were significantly higher in LC cows and lower in FC and IO cows than in HC cows (8.30 ± 2.08, 0.33 ± 0.14, and 0.41 ± 0.18 vs. 5.36 ± 0.58 ng/mL, *P* < 0.05) (Figure 2G). Plasma IGF-1 levels were significantly lower in FC and IO than in HC cows (79.37 ± 13.37, and 28.25 ± 1.47 vs. 104.74 ± 4.65 ng/mL, *P* < 0.05) (Figure 2H).

**Table 2:** Kruskal-Wallis test of plasma energy metabolites, liver function indicators, minerals and hormones among the four groups (*n* = 76).

**Parameters**	**Mean**	**SD**	**0**	**25%**	**50%**	**75%**	**100%**	**χ^2^**	***P*-value**
β-hydroxybutyrate (mmol/L)	0.82	0.36	0.3	0.5	0.9	1.03	1.6	3.38	0.337
NEFA (mmol/L)	0.5	0.15	0.26	0.41	0.46	0.57	0.94	18.963	<0.001
Glucose (mmol/L)	3.13	0.31	2.57	2.93	3.17	3.25	3.86	3.333	0.343
Triglycerides (mmol/L)	0.1	0.03	0.05	0.08	0.1	0.11	0.25	0.825	0.844
Total cholesterol (mmol/L)	3.85	1.27	1.74	2.85	3.35	4.97	6.99	1.763	0.623
Urea nitrogen (mmol/L)	4.53	0.91	2.86	3.86	4.48	5.13	6.31	3.827	0.281
Aspartate aminotransferase (U/L)	57.21	25.53	21	38.25	56	73.25	154	4.446	0.217
ALT (U/L)	17.26	7.56	6	11	16.5	22.25	44	8.094	0.044
γ-glutamyltransferase (U/L)	23.47	8.18	10	18	23	30	39	3.609	0.307
Total protein (g/L)	55.04	14.86	26.2	42	58.75	66.5	78.3	1.893	0.595
Albumin (g/L)	27.46	6.83	11.1	22	28.85	34.33	37.7	2.059	0.56
Globulin (g/L)	27.59	8.8	15.13	19.8	28.63	32.84	46.05	1.243	0.743
Total bilirubin (μmol/L)	3.65	0.72	2.4	3.08	3.55	4.23	5.2	4.478	0.214
Calcium (mmol/L)	2.07	0.18	1.53	2.01	2.1	2.18	2.33	11.424	0.01
Phosphorus (mmol/L)	1.86	0.28	1.32	1.74	1.84	2.03	2.5	15.354	0.002
Magnesium (mmol/L)	1.16	0.11	0.97	1.09	1.18	1.25	1.34	7.466	0.058
Estradiol (pg/mL)	14.98	6.06	5.61	9.09	14.88	19.77	27.68	14.067	0.003
Progesterone (ng/mL)	4.51	3.9	0.1	0.65	5.03	6.66	18.57	19.228	<0.001
Insulin (ng/mL)	14.74	6.2	4.68	9.54	13.93	17.45	34.44	4.555	0.207
Growth hormone (ng/mL)	44.91	6.17	34.96	40.97	44.03	49.6	57.87	0.361	0.948
IGF-1 (ng/mL)	93.54	34.03	25.7	76.58	99.45	119.25	154	12.17	0.007

*Note: NEFA, non-esterified fatty acids; ALT, alanine aminotransferase; IGF-1, insulin-like growth factor 1*.

**Figure 3:**
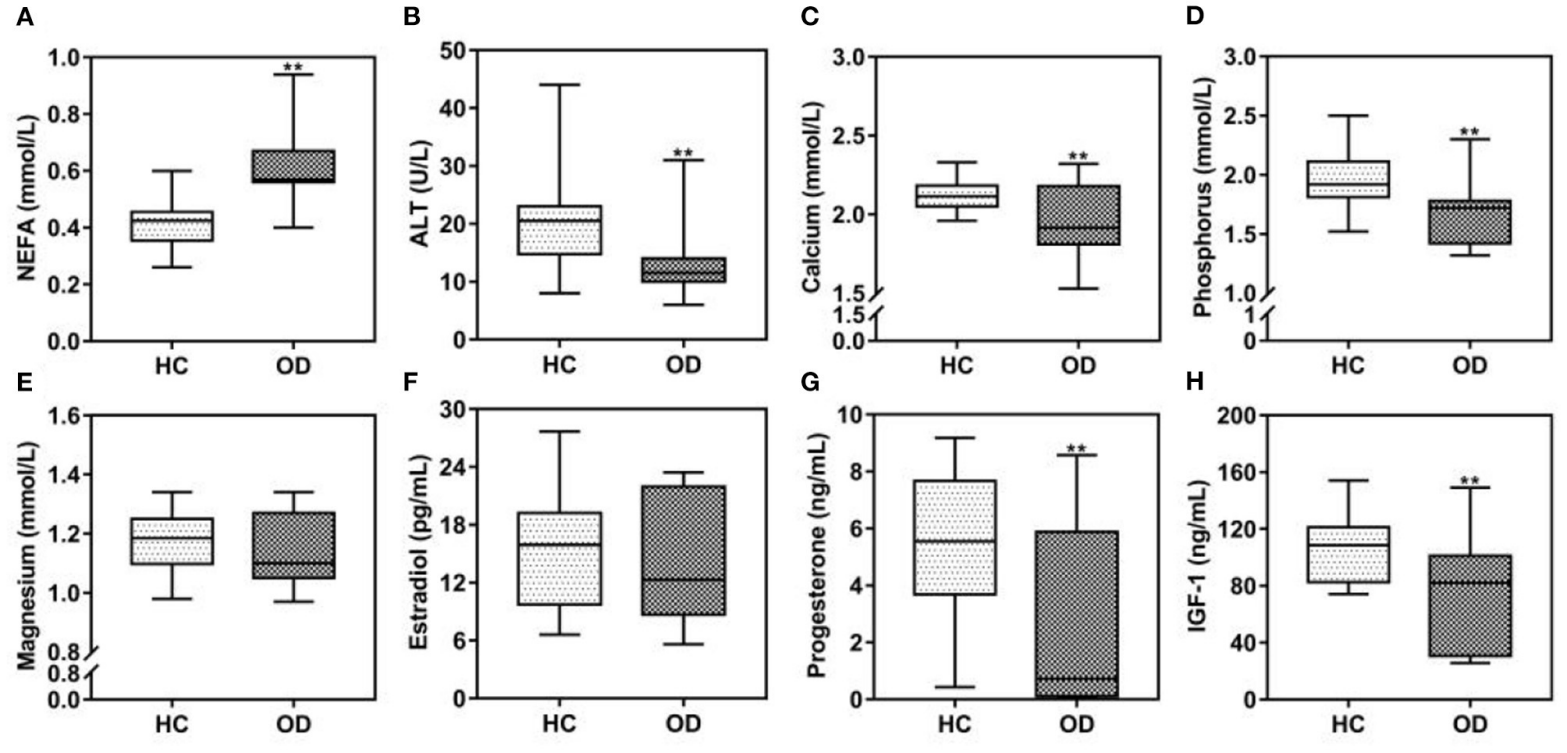
**(A–H)** Comparison of plasma biochemical indicators between the ovarian diseases (OD, *n* = 54) group and healthy control (HC, *n* = 22) group at 3 weeks postpartum. Note: ^**^P < 0.01 vs. healthy control group. NEFA, non-esterified fatty acids; ALT, alanine aminotransferase; IGF-1, insulin-like growth factor 1.

**Figure 4:**
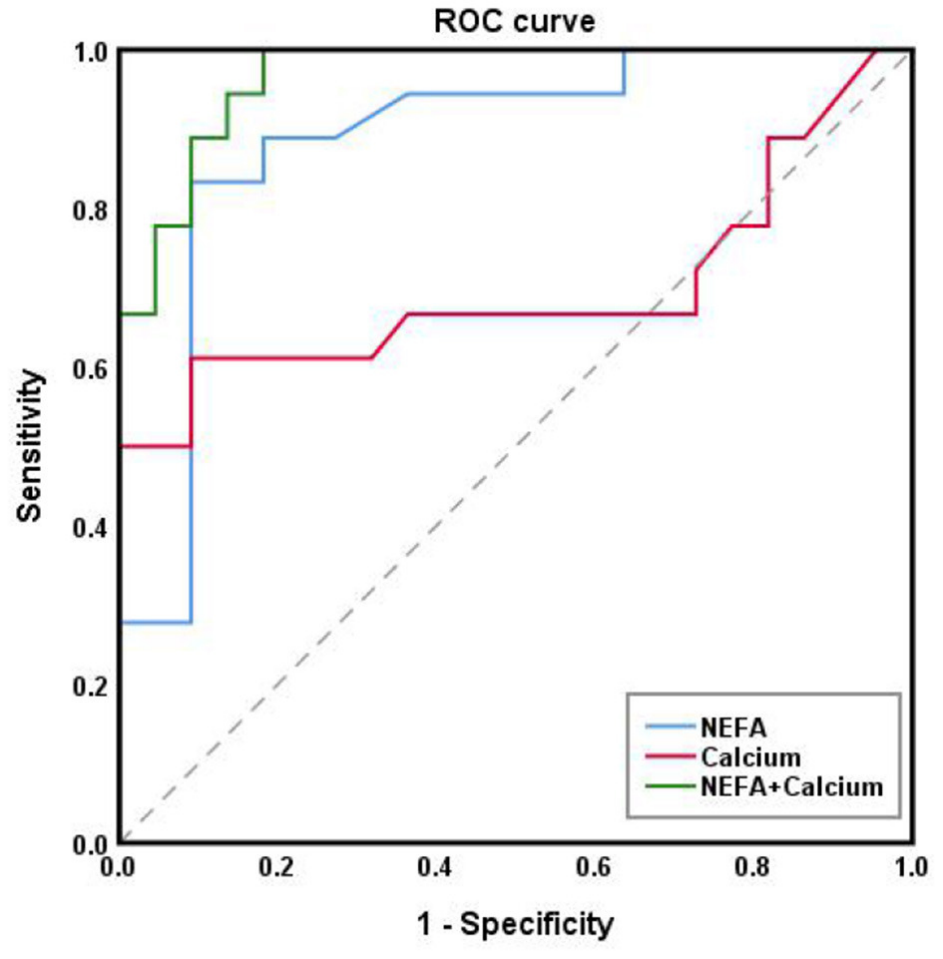
ROC area under the curve of NEFA and calcium in dairy cows with ovarian diseases.

From here on, we refer to the three diseases collectively as “ovarian diseases.” Statistical comparison between two groups was performed using the Mann–Whitney U-test. Compared to healthy controls, cows with ovarian diseases had higher (*p* < 0.01) NEFA and lower (*P* < 0.05) ALT, calcium, phosphorus and IGF-1 concentrations (Figure 3). Spearman correlation analysis showed that plasma NEFA level was positively correlated (*P* < 0.01) with ovarian diseases, while plasma ALT, calcium, phosphorus, progesterone, and IGF-1 levels were negatively correlated (Table 3, *P* < 0.01). With each of them, a binary logistic regression analysis was performed. Notably, only two of these parameters (NEFA and calcium) correlated with ovarian diseases. To obtain the early warning indicators of ovarian diseases, we applied ROC curve analysis to evaluate the accuracy of our test. ROC curve analysis indicated that a NEFA value of 0.50 mmol/L discriminated diseased from healthy cows with an area under the curve of 0.886, sensitivity of 83.3% and specificity of 90.9% (Figure 4 and Table 4). Similarly, the sensitivity and specificity of calcium in predicting ovarian diseases were 61.1 and 90.9%, respectively; when the cutoff value of calcium was settled at 2.02 mmol/L, the area under the curve was 0.697 (Figure 4 and Table 4). Furthermore, this analysis also showed that NEFA combined with calcium for the diagnosis was prior than the single indicator. Specifically, this model performance increased as calcium was added, with an area under the curve of 0.967, sensitivity of 100%, and specificity of 81.8% (Figure 4 and Table 4).

**Table 3:** Correlation between plasma biochemical indexes and ovarian diseases in dairy cows (*n* = 76).

**Parameters**	**Mean**	**SD**	***R*-Value**	***P*-Value**
NEFA (mmol/L)	0.55	0.16	0.612^**^	<0.001
ALT (U/L)	15.39	6.95	−0.426^**^	<0.001
Calcium (mmol/L)	2.01	0.21	−0.310^**^	0.006
Phosphorus (mmol/L)	1.76	0.3	−0.497^**^	<0.001
Progesterone (ng/mL)	3.69	4.31	−0.372^**^	<0.001
IGF-1 (ng/mL)	81.9	39.73	−0.348^**^	0.002

*Note: R is the spearman correlation coefficient, R > 0 means positive correlation; R < 0 means negative correlation*.

** * indicates extremely significant correlation with ovarian diseases (P < 0.01). NEFA, non-esterified fatty acids; ALT, alanine aminotransferase; IGF-1, insulin-like growth factor 1*.

**Table 4:** The cutoff point, sensitivity, specificity, standard error and area under the ROC curve of NEFA and Calcium diagnosed by ovarian diseases in dairy cows.

**Parameters**	**Cutoff point (mmol/L)**	**Sensitivity (%)**	**Specificity (%)**	**AUC**	**SEM**	***P*-Value**
NEFA	0.5	83.3	90.9	0.886 (0.794–0.979)	0.047	<0.001
Calcium	2.02	61.1	90.9	0.697 (0.581–0.813)	0.059	0.007
NEFA + Calcium	–	100	81.8	0.967 (0.929–1.000)	0.02	<0.001

*Note: NEFA, non-esterified fatty acids; AUC, area under the ROC curve*.

## Discussion

Ovarian diseases are one of the most common ovarian dysfunctions in dairy cattle, which can lead to a considerable economic loss through its high incidence and can reduce the reproductive performance (6). Although several studies have reported that ovarian diseases are related to alterations in metabolites and hormonal factors (35), the mechanisms involved in the development of ovarian diseases are yet to be elucidated along with the contribution of other possible pathogenic determinates. We therefore compared the plasma energy metabolic, liver function, mineral and hormone levels (8 wk postpartum) between the FC cows, LC cows, and IO cows and HC cows, respectively. Furthermore, earlier diagnosis, especially before 3 weeks postpartum, may facilitate access to interventions that improve ovarian diseases. To obtain biomarkers for early diagnosis of the diseases, we evaluated the early warning effects of plasma biochemical indicators (3 wk postpartum) on cows with ovarian diseases, including FC, LC and IO.

Follicular cysts are anovulatory follicular structures (11). Its etiology is not fully understood, but hormonal and metabolic abnormalities are recognized as important causes of FC (36). Cows diagnosed with FC have a dysfunctional hypothalamic-pituitary-ovarian axis signature that includes parabasal concentration of progesterone (<1 ng/mL), increased peripheral estradiol levels, increased lutenizing hormone pulse frequency and amplitude, and reduced lutenizing hormone and follicle-stimulating hormone receptors that translate into a lack of lutenizing hormone surge and ovulation (36). Consistent with this, we observed that, as expected, FC cows in the current study had higher peripheral estradiol levels and lower progesterone levels (0.33 ± 0.14 vs. 5.36 ± 0.58 ng/mL) compared with HC cows. Moreover, we also observed that FC cows had higher plasma levels of NEFA and BCS loss in early lactation than HC cows. These observations are consistent with several previous studies (36–38). The increased plasma NEFA levels and higher BCS loss in FC cows suggests greater adipose tissue mobilization during the NEB in early lactation (39). Numerous studies showed the adverse effects of NEFA for granulosa cell proliferation and survival, steroidogenesis, and follicular development and oocyte maturation (40–43). Therefore, it is reasonable to speculate that the increased plasma NEFA concentrations found in FC cows in the current study may damage the granulosa cell function and steroidogenesis. Although the pathogenesis and mechanism of cyst formation are not fully understood, it has been proposed that the IGF system could play an essential role, as it is a key intraovarian regulator (44). Beam et al. (45) reported that follicular competence early postpartum was associated with higher plasma IGF-1. Our findings for IGF-1 in FC and HC cows were consistent with a previous study that reported reduced plasma levels of IGF-1 in FC cows when compared to HC cows (37). It is well-known that ALT is the most widely used biomarker to detect liver disease in clinical practice (46). Increased levels of circulating ALT may reflect the underlying liver pathology. A recent study that revealed half of FC cows with liver disorders, such as fatty liver and hepatitis (47). This finding suggests that liver disorders are well-connected to development of FC in dairy cows and that steroid hormone metabolism is delayed, resulting in the formation of FC (47). On the contrary, we observed that FC cows had lower plasma ALT levels, and we postulate that an enhanced hepatic lipid mobilization accounts for this phenomenon (48). Altogether, these previous findings along with ours, indicate that the formation of FC is associated with an increased fat mobilization and the disorder of steroidogenesis caused by NEB in early lactation.

The formation of LC is attributed to luteinnization of unovulated follicles, its pathogenesis still remains unclear. It is generally believed that LC is related to abnormal corpus luteum function. Previous research suggested that cows with LC might tend to be in anoestrus as the higher amount of progesterone secreted by this luteinized structure may change the pattern of gonadotrophin's secretion (13). Douthwaite and Dobson reported that the mean plasma progesterone concentration was higher in the cows with LC than in those with HC (49). Furthermore, the mean plasma progesterone concentration of a cow with a LC has been reported to be 3.6 ng/mL (50), with a range from 3.0 to 10.4 ng/mL (51). These values are similar to values reported in our current study. Cows that had delayed commencement of luteal activity had high NEFA values at 1-week postpartum (52). In addition, Both the areas occupied by endothelial and by steroidogenic cells were negatively correlated with the blood concentration of NEFA (53). These findings are similar ours and lend further support to a causal role of NEFA in LC development. High concentration of plasma NEFA and low milk production in LC cows are related to NEB in early lactation (54). An early study by Davis et al., suggested that Ca^2+^ and phospholipid enhanced phosphorylation of endogenous luteal cytosol protein, while low concentration of Mg^2+^ inhibited the phosphorylation of target protein (55). The same study also showed that a phospholipid-sensitive, Ca^2+^-dependent protein kinase might provide an important link between hormonally-induced changes in phospholipid metabolism and corpus luteum function. In the present study, we found that LC cows had lower plasma levels of calcium, phosphorus, and magnesium than HC cows, suggesting phosphorylation disorder was present in LC cows. Altogether, these previous findings along with ours, suggest that the formation of LC is related to the disorder of steroidogenesis caused by NEB in early lactation and the disorder of phosphorylation caused by mineral deficiency. Importantly, both mechanisms would lead to abnormal corpus luteum function.

Inactive ovaries are driven by a loss of periodic follicular activity and transient ovarian dysfunction (56). Nutritional status is generally recognized as a significant contributing factor to inactive ovaries, due to a close link between postpartum ovarian activity and energy balance (57, 58). Our current result shows that cows with inactive ovaries have a significant lower BCS than healthy control cows. This is consistent with another report showing that low BCS is associated with inactive ovaries (59). Low levels of estradiol and progesterone in plasma were also observed in cows with inactive ovaries but not in healthy controls. This fits the known hormonal characteristics of inactive ovaries (60). The plasma concentration of NEFA characterizes the magnitude of fat mobilization (61). Meanwhile, an increase in circulating NEFA is often an accurate indicator of NEB in dairy cows (62). IGF-1 plays an important role in gonadotropin-induced folliculogenesis, ovarian steroidogenesis and corpus luteum function. It also modulates pituitary and hypothalamus function (63). Zulu et al. reported negative association of serum IGF-1 with NEFA but positive association with estradiol (37). The same study also reported that NEFA was higher and IGF-1 was lower in inactive ovary than in normal cows. These results is consistent with our observations. Interestingly, cows with inactive ovaries had low ALT levels similar to that observed in FC cows. Our isolated finding should be interpreted with caution. The direct role of calcium and phosphorus in the inactive ovaries of cows was undescribed so far, but we observed that the plasma calcium and phosphorus levels were significantly lower in cows with inactive ovaries compared with healthy control cows. Notably, a recent study demonstrated that hypocalcemic and hypophosphatemic female mice developed infertility accompanied by decreased estradiol and progesterone levels, elevated follicle-stimulating hormone and luteinizing hormone levels, defects in follicular development and corpus luteum formation, uterine hypoplasia, and decreased ovarian expression of angiogenic factors (64). It is reasonable to speculate that calcium and phosphorus play a role in the development of inactive ovaries. Collectively, these results indicate that the onset of inactive ovaries may not only be relevant for an increased fat mobilization caused by NEB in early lactation, but also for the low levels of plasma calcium and phosphorus. However, the synergy mechanisms underlying the both in inactive ovaries remain to be further investigated.

As mentioned in the introduction, many biomarkers of energy status such as β-hydroxybutyrate, NEFA, calcium and BCS have been used to evaluate postpartum diseases in dairy cows (65). However, little is known about the early warning and assessment of ovarian diseases in dairy cows. Although blood biomarkers at the time of ovarian diseases can be used to monitor diseases themself, it is too late to implement appropriate interventions. Previous studies have addressed this same question. Ospina et al. reported lower sensitivity and specificity of NEFA measured pre-partum (threshold of ≥0.39 mmol/L) compared with post-partum (threshold of ≥0.57 mmol/L) for detection of any of the following diseases: displaced abomasum, clinical ketosis, metritis, or retained placenta (66). Pre-partum sensitivity and specificity were 48 and 69%, while post-partum sensitivity and specificity were 75 and 61% (66). Calcium concentrations can be used to identify hypocalcemia, which occurs when calcium levels are below the normal reference range of 8.5–10 mg/dl (67). Calcium serum levels below 9.4 mg/dl measured 1-week pre-partum had very low sensitivity at 35% for displaced abomasum, while specificity was 71% (25). In this study, we found that NEFA and calcium also predict ovarian diseases in dairy cows. A higher sensitivity and specificity of NEFA measured (threshold of ≥0.50 mmol/L) compared with calcium (threshold of <2.02 mmol/L) for the prediction of ovarian diseases. NEFA sensitivity and specificity were 83.3 and 90.9%, while calcium sensitivity and specificity were 61.1 and 90.9%. Furthermore, the sensitivity (100%) and specificity (81.8%) were greater when a combined NEFA and calcium approach was applied. Similarly, the area under the ROC curve for the prediction of ovarian diseases was 0.967, which was higher than those regarding NEFA (0.886) and calcium (0.697). The risk of ovarian diseases onset increases for concentrations above the NEFA thresholds and/or below the calcium thresholds. Since most ovarian diseases incidence occurs at 8 weeks postpartum, this allows for approximately 30 days to implement interventions to prevent disease development in at-risk cattle. Interventions may include improving environmental factors, nutrition, or transitional treatments.

## Conclusion

In summary, we found that ovarian diseases were associated with an increased fat mobilization and the disorder of steroidogenesis caused by early postpartum NEB and the disorder of phosphorylation duo to mineral deficiency. Importantly, higher plasma NEFA levels and lower calcium levels could be used to predict an increased risk of ovarian diseases in dairy cows. In particular, NEFA could be applied jointly with calcium to achieve a higher prediction performance. To the best of our knowledge, this is the first study that has reported models predictive of early lactation disease incidence at the cohort level using aggregate biomarkers measured at 3 wk postpartum. Future work could include validating the models in studies with larger sample sizes and building separate models for different ovarian diseases or groups of ovarian diseases. Our results have important practical implications. Earlier and more accurate disease prediction will result in the ability to implement earlier interventions, thereby potentially reducing the incidence of ovarian diseases.

## Data Availability Statement

The original contributions presented in the study are included in the article/supplementary material, further inquiries can be directed to the corresponding author.

## Ethics Statement

The animal study was reviewed and approved by Ethics Committee on the Use and Care of Animals of Heilongjiang Bayi Agricultural University.

## Author Contributions

YS: analyzed the data and wrote the manuscript. CXia and CXu: designed the study and revised the manuscript. ZW and YB: participated in the acquisition of the data. JC and HY: performed the laboratory analysis. All authors contributed to the article and approved the submitted version.

## Funding

The Key Project of Natural Science Foundation of Heilongjiang Province of China (ZD2021C006).

## Conflict of Interest

The authors declare that the research was conducted in the absence of any commercial or financial relationships that could be construed as a potential conflict of interest.

## Publisher's Note

All claims expressed in this article are solely those of the authors and do not necessarily represent those of their affiliated organizations, or those of the publisher, the editors and the reviewers. Any product that may be evaluated in this article, or claim that may be made by its manufacturer, is not guaranteed or endorsed by the publisher.
